# Oocyte maturation triggering in high responders in IVF treatment: a systematic review and network meta-analysis

**DOI:** 10.3389/fendo.2026.1669781

**Published:** 2026-04-02

**Authors:** Yusuf Beebeejaun, Timothy Copeland, James M. N. Duffy, Ippokratis Sarris, Marian Showell, Sesh K. Sunkara

**Affiliations:** 1 Department of Women’s and Children’s Health, Faculty of Life Sciences and Medicine, King’s College London, London, United Kingdom; 2 Department of Clinical Neuroscience Karolinska Institute, Solna, Sweden; 3 Centre for Reproductive Medicine, St Bartholomew’s Hospital, London, United Kingdom; 4 King’s Fertility, Fetal Medicine Research Institute, London, United Kingdom; 5 Department of Obstetrics and Gynecology, University of Auckland, Auckland, New Zealand

**Keywords:** IVF, trigger, clinical pregnancy rate (CPR), ovarian hyper stimulation syndrome, network meta analyses, systematic review

## Abstract

**Objective:**

The aim of this study was to compare and rank the efficacy and safety of four final oocyte maturation trigger strategies—human chorionic gonadotropin (hCG), gonadotropin-releasing hormone agonist (GnRHa), dual, and double trigger—in predicted high responders undergoing *in vitro* fertilization (IVF) with GnRH antagonist protocols, using a network meta-analysis (NMA) approach.

**Methods:**

A systematic search of MEDLINE, EMBASE, CENTRAL, clinical trial registries, and the Cochrane Database of Systematic Reviews was conducted through December 2024. Eligible studies were randomized controlled trials (RCTs) including high responders, defined by elevated antral follicle count, anti-Müllerian hormone, or estradiol levels. Studies using GnRHa triggers followed by fresh embryo transfer were included only if intensive luteal phase support was provided. Oocyte donation cycles, quasi-randomized designs, and trials lacking outcome data were excluded. Data extraction and risk of bias assessment were independently conducted by two reviewers. Study integrity was evaluated using the TRACT checklist. NMA was performed in STATA (v16), and treatment ranking was based on Surface Under the Cumulative Ranking curve (SUCRA).

**Results:**

Seven high-quality RCTs comprising 632 women were included. There were no significant differences in the number of oocytes retrieved between GnRHa and hCG triggers (mean difference [MD] 1.08, 95% CI –1.06 to 3.22), dual and hCG (MD 0.61, 95% CI –1.53 to 2.74), or GnRHa and dual (MD 1.08, 95% CI –1.06 to 3.22). Similarly, there were no significant differences in mature oocyte yield, clinical pregnancy rate (CPR), or miscarriage rate across comparisons. However, GnRHa trigger significantly reduced the risk of moderate to severe ovarian hyperstimulation syndrome (OHSS) compared with hCG (RR 0.23, 95% CI 0.07–0.82). There were no significant differences in OHSS risk between dual and hCG (RR 0.28, 95% CI 0.05–1.64) or between GnRHa and dual (RR 0.28, 95% CI 0.05–1.64).

**Conclusion:**

GnRHa, hCG, and dual triggers demonstrate similar efficacy in terms of oocyte yield, maturity, and clinical pregnancy rates in predicted high responders. The GnRHa trigger, however, offers a superior safety profile by significantly lowering the risk of OHSS. Larger multicenter RCTs are required to evaluate live birth outcomes and the potential role of the double trigger in this population.

**Systematic review registration:**

https://www.crd.york.ac.uk/prospero/, identifier CRD42022351423.

## Introduction

Final oocyte maturation is physiologically initiated by the pre-ovulatory surge of luteinizing hormone (LH) (1, 2) which initiates a cascade within granulosa cells and oocytes, loss of oocyte-cumulus gap junctions, meiotic resumption to metaphase II, and granulosa-cell luteinization, while the concomitant progesterone rise reinforces estradiol’s positive feedback to elicit the mid-cycle FSH peak (3, 4), The associated physiological FSH surge, in turn, contributes to up-regulation of LH receptors on granulosa cells and synthesis of a hyaluronic-acid matrix, enabling cumulus expansion and dispersion so that the oocyte–cumulus complex becomes free-floating within the antrum (5–8).

Traditionally, in a cycle of *in-vitro* fertilization (IVF), human chorionic gonadotropin (hCG) has been a gold standard substitute for the endogenous LH surge due to its structural similarity to LH and its ability to bind and activate the LH/hCG receptors thereby triggering the resumption of meiosis (9). Standard dosing includes urinary hCG 5,000–10,000 IU subcutaneously/intramuscularly or recombinant hCG 250 µg subcutaneously (≈6,500 IU bioactivity), administered ~36 hours before oocyte retrieval (8). However, the extended luteotropic activity caused by hCG administration (4, 10), combined with elevated levels of estradiol and progesterone, can result in ovarian hyperstimulation syndrome (OHSS), particularly in high responders, requiring a postponement of embryo transfer (11–13).

In 1990, Gonen et al. (14) demonstrated that ovulation can also be induced using a gonadotropin-releasing hormone (GnRH) agonist trigger. By displacing the GnRH antagonist from the receptor, the GnRHa trigger causes a flare-up of both LH and FSH, similar to the natural cycle, and therefore regarded as a more physiological trigger for ovulation (5, 11). In 2000, Itskovitz-Eldor et al. (15) reported the first series of patients at high risk of developing severe OHSS who underwent controlled ovarian hyperstimulation (COH) using a GnRH antagonist protocol with a GnRHa trigger for final follicular maturation. While 50% of the patients achieved conception, none developed clinical or biochemical signs of OHSS. This is because, unlike hCG, GnRH agonists have a shorter half-life, leading to rapid luteolysis and significantly reducing OHSS risk (16). As a result, the use of GnRHa trigger, within a GnRH antagonist cycle, has become a cornerstone in achieving OHSS-free IVF practice (16).

Building on these observations, multiple studies have compared hCG with GnRHa for final oocyte maturation in IVF (17, 18). Across reports, the number of oocytes retrieved, the proportion of metaphase II oocytes, and the yield of top-quality embryos were generally comparable and in several studies favored the GnRHa trigger (19, 20). However, their use has been associated with significantly lower clinical pregnancy rates and higher first-trimester pregnancy losses (12, 21). This was due to the associated luteal phase insufficiency, marked by lower progesterone levels, which can compromise the peri-implantation period and result in higher early pregnancy loss rates following fresh embryo transfer (22–27). Various strategies were therefore explored to optimize reproductive outcomes to modify luteal phase support by enhancing corpus luteum function (25, 28). One proposed approach involves the co-administration of GnRHa with a standard hCG bolus (5,000–10,000 IU) before oocyte retrieval to enhance final follicular maturation, with the aim of improving oocyte competence, embryo quality, and overall IVF outcomes while addressing the luteal insufficiency associated with GnRHa administration alone (11, 28).

The dual trigger involves the co-administration of GnRH-a with low-dose hCG (≈1,000–2,500 IU) given simultaneously at trigger (29) and the double (staggered) trigger administers GnRH-a ~40 h and hCG ~34 h before retrieval, a timing variant conceived to optimize final maturation and cumulus expansion in selected contexts (24). Their mechanisms and clinical advantages however differ. The dual trigger believed to more closely mimic the physiological mid-cycle surge, has been reported to result in improved oocyte maturation, embryo quality, and pregnancy outcomes without increasing the risk of OHSS (30, 31) while the extended LH surge provided by the double trigger staggered approach has been reported to result in an improvement in follicular synchronization, thereby enhancing oocyte yield and maturity in patients with previous suboptimal responses or empty follicle syndrome (32, 33).

Although prior individual randomized controlled trials have performed pairwise comparison assessing the effectiveness of individual protocols such as hCG, GnRHa, dual, or double triggers to each other, there has not been a comprehensive analysis simultaneously evaluating all four trigger protocols in high responder patients. Evidence syntheses have also typically been pairwise, often mixing heterogeneous patient populations or not isolating predicted high responders. As a result, there is a lack a comprehensive, hierarchical comparison of all four trigger protocols specifically in high-risk patients and there remains a lack of consensus on the optimal triggering protocol for high responders where trigger choice has direct implications for safety, embryo transfer strategy, and time to pregnancy.

In light of these considerations, the objective of our study is to address this gap in research literature and conduct a systematic review of existing high integrity evidence and perform a network meta-analysis to simultaneously assess the efficacy and safety of the hCG trigger alone, GnRHa trigger alone, dual trigger, and double trigger protocols in predicted high responder patients.

## Methods

### Search and study selection

We adhered to the PRISMA extension statement for reporting systematic reviews with network meta-analyses of healthcare interventions in reporting this systematic review (34). The protocol was registered on PROSPERO: International Prospective Register of Systematic Reviews, with registration number CRD42022351423.

We conducted a systematic search, from their inception up to December 30, 2024, across several databases including MEDLINE, EMBASE, Cochrane Database of Systematic Reviews and Cochrane Central Register of Controlled Trials (CENTRAL) which included trials registered on clinicaltrials.gov as well as the trial registry of the World Health Organization and the Cochrane Gynaecology and Fertility specialised register. Two authors (Y.B. and J.M.N.D) examined the identified studies independently for compliance with inclusion criteria and selected eligible studies. They resolved disagreements by discussing with a third author (S.S.) and documented the selection process with a ‘PRISMA’ flowchart.

The search was specifically focused on RCTs which compared different triggers for final oocyte maturation—hCG, GnRHa, dual trigger, or double trigger—in patients defined as high responders and undergoing ovarian stimulation for IVF/ICSI within GnRH antagonist cycles. Both free text and index terms were utilized for the search, as detailed in **Supplementary Appendix 1**.

### Study eligibility

Only randomized trials were eligible. Cross-over, quasi-randomized, and non-randomized trials were excluded, as well as studies involving oocyte donation transfer cycles. Studies were restricted to patients predicted to be high responders defined as per at least one of the following criteria: a normal FSH level with a history of PCOS as per Rotterdam criteria (35, 36), number of antral follicles more than 20, anti-Mullerian hormone level >3.5 ng/mL, number of follicles >18 mm more than 20, or estradiol level more than 4000 pg/mL (37). These definitions were used in place of any newer criteria which were not adopted when the previous RCTs were published. Where necessary, additional trial details or protocols were sought to determine potential study eligibility.

Studies involving GnRH antagonist protocols and utilizing GnRHa trigger alone followed by fresh transfers were included only if they incorporated intensified luteal progesterone support (LPS) according to the previously defined criteria and consisted of one of the following: micronized progesterone vaginally, estradiol supplementation trans-dermally or vaginally (19, 23, 26, 38) daily low dose hCG administration during the luteal phase without exogenous progesterone (39), a single bolus of hCG given after oocyte retrieval (40, 41), recombinant LH (Luveris, Merck-Serono) starting on the day of oocyte retrieval up to day 10 after oocyte retrieval (32).

### Risk of bias, trustworthiness, and overall certainty of evidence assessments

Two authors (Y.B. and J.M.N.D.) independently gathered data from the included studies and assessed the risk of bias utilizing the Cochrane Collaboration’s risk of bias assessment tool. Any discrepancies were resolved through discussion with a third author (S.S.). We evaluated selection bias, performance bias, detection bias, attrition bias, reporting bias, and other biases.

Identified RCTs were further evaluated for trustworthiness using the Trustworthiness in Randomized Controlled Trials (TRACT) checklist (42) which assessed 19 items organized into seven domains that are applicable to every RCT: 1) Governance, 2) Author Group, 3) Plausibility of Intervention Usage, 4) Timeframe, 5) Drop-out Rates, 6) Baseline Characteristics, and 7) Outcomes [32]. Only studies that met high integrity standards according to the TRACT checklist were included. Authors were contacted for clarification regarding these discrepancies and reasons for exclusion included lack of prospective clinical trial registration. The overall quality of evidence was also assessed using the web application Confidence in Network Meta-Analysis (CINeMA), which is based on the Grading of Recommendations, Assessment, Development, and Evaluations framework (43). (**Supplementary Table 1**).

### Outcomes

In addition to reporting the recommended core outcome set, developed for infertility research (44, 45), such as clinical pregnancy, live birth and pregnancy loss, we also included non-core outcome outcomes such as number of oocytes collected, number of mature oocytes retrieved and rate of OHSS per patient randomized per stimulation cycle. We aimed to extract data for other core outcomes such as gestational age at delivery; birthweight; neonatal mortality; major congenital anomaly and time to live birth but these were not reported.

Clinical pregnancy was defined as a pregnancy diagnosed by ultrasonographic visualization of one or more gestational sacs or definitive clinical signs of pregnancy (46). Live birth was defined as a birth in which a fetus is delivered with signs of life after complete expulsion or extraction from its mother (46). Miscarriage was defined as spontaneous loss of an intrauterine pregnancy prior to twenty-two completed weeks of gestational age (46). A mature oocyte was defined as an oocyte at metaphase of meiosis II, exhibiting the first polar body and with the ability to become fertilized (46). Ovarian hyperstimulation was defined as an exaggerated systemic response to ovarian stimulation characterized by a wide spectrum of clinical and laboratory manifestations (46).

As outlined in our PROSPERO registration, live birth was initially identified as a primary outcome. However, due lack of reporting and available evidence, it was not possible to perform a comprehensive network meta-analysis on this outcome. Our analysis focused on laboratory outcome (oocyte yield, mature oocyte yield), clinical outcomes (clinical pregnancy and miscarriage rates) and rate of OHSS.

### Data synthesis and analysis

Statistical analysis for pairwise was performed using STATA version 16 (STATA Corp., College Station, TX, USA). We used network plots to visually represent all direct comparisons among the randomized controlled trials (RCTs) included in our study (47). To assess potential inconsistency, we employed a design-by-treatment interaction model (48). If no inconsistency was identified, we performed pairwise and network meta-analyses using a random-effects model in STATA, utilizing the ‘network’ command for network meta-analysis and other STATA commands for visualizing and reporting results (49). Effect estimates are presented as risk ratios (RR) for dichotomous outcomes and mean differences (MD) for continuous outcomes, with 95% confidence intervals (CI).

We used the Surface Under the Cumulative Ranking (SUCRA) methodology to establish a hierarchy among the interventions considered in our study (50). Notably, a higher SUCRA value indicates a more favorable treatment rank. For example, if the SUCRA for treatment A is 100%, all other treatments are considered inferior to A, indicating A as the best.

In addition, a *post-hoc* power analysis was carried out to assess the detectability of the observed differences given the sample sizes available. This analysis was performed using G*Power (43), employing a two-tailed z-test for the difference between two independent proportions. We set the significance level (α) at 0.05 and used the actual sample sizes from our included studies as the basis for the analysis. The analysis computed the critical z-value and the achieved power (1 - β error probability), which reflects the probability of correctly detecting an effect of the magnitude observed if it truly exists. A *z*-value exceeding ±1.96 was considered statistically significant at the 5% level (two-tailed), indicating that the observed difference between intervention groups would be unlikely to have occurred by chance.

## Results

### Study selection and characteristics

**Figure 1:**
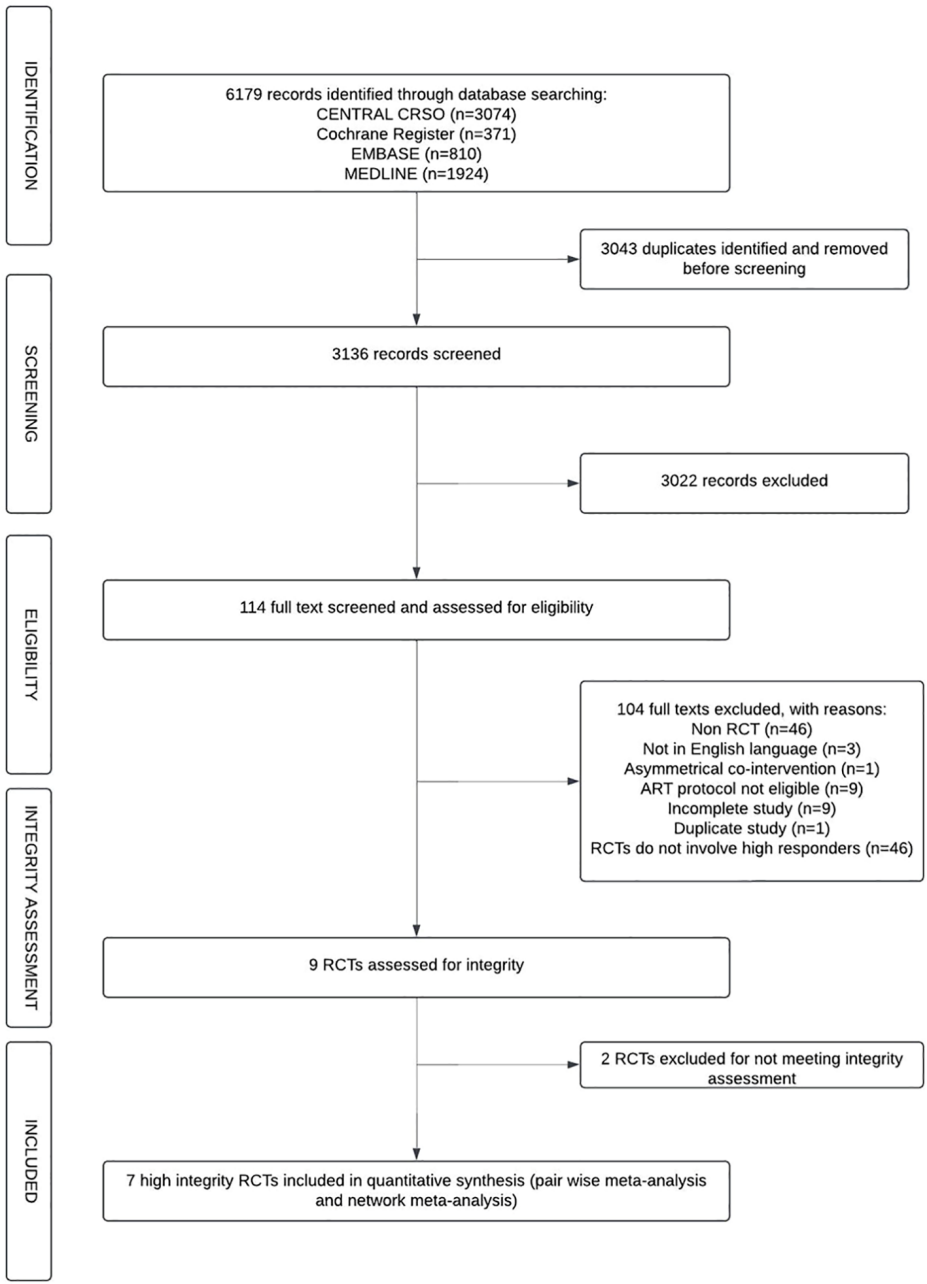
PRISMA 2020 flow diagram showing identification, screening, eligibility, integrity assessment, and inclusion of randomised controlled trials for the systematic review and network meta-analysis.

The initial electronic database search yielded 6,179 records. After excluding 3,043 duplicate records, 3,136 titles and abstracts were screened for potential inclusion. 3,022 were further excluded and 114 full text articles were further assessed for eligibility. Nine RCTs, reporting data from 961 participants met our inclusion criteria for integrity assessment (**Figure 1**) (22, 23, 37, 38, 51–55).

Reports with concerns about trustworthiness included two full-text publications. We contacted the authors of these studies for clarification and they were ultimately excluded from the final analysis due to a lack of prospective clinical trial registration and lack of information to assess discrepancies of  > 15% between the intended sample size in the trial registration compared to the actual sample size achieved in the trial (53, 55).

Seven RCTS, representing 632 IVF/ICSI participants were finally included in our systematic review, pair wise meta-analysis and network meta-analysis (22, 23, 38, 51, 52, 54, 56). Publication dates ranged from 2006 to 2022, four studies published within the last 10 years (37, 38, 41, 54), conducted in six countries. The study characteristics of the included studies are listed in **Table 1**.

**Table 1:** Summary of included randomised controlled trials comparing ovulation-trigger strategies in high-responder IVF patients.

Author (year)	Country	Design/population	Trigger 1 (intervention)	Dose/timing	Outcome	N/mean and SD	Total number of participant in arm 1	Trigger 2 (control)	Dose / Timing	Outcome	n	Total number of participant in arm 2
Aghahosseini et al. (2017) (37)	Iran	RCT – PCOS / high risk	Dual trigger	Buserelin 0.5 mL + hCG 1 500 IU (simultaneous administration)	CPR	18	40	GnRHa only	Buserelin 0.5 mL + hCG 1 500 IU 35 h later for luteal support	CPR	19	40
					Number of oocytes	26.5 (8.9)				Number of oocytes	24.4 (8.5)	
					OHSS	13				OHSS	5	
Babayof et al. (2006) (22)	Israel	RCT – PCO patients	GnRHa trigger	Triptorelin 0.2 mg (Decapeptyl)	OHSS	0	15	hCG trigger	250 IU Ovitrelle	OHSS	4	13
					Miscarriage	2				Miscarriage	2	
					Number of mature oocytes	18 (2.9)				Number of mature oocytes	16 (2.2)	
Elgindy et al. (2018) (41)	Egypt	RCT – High responder ICSI	GnRHa trigger	Triptorelin 0.3 mg + 1 500 IU hCG at retrieval for luteal support	OHSS	10	95	hCG trigger	10 000 IU + standard luteal support	OHSS	23	95
					CPR	51				CPR	53	
					Number of mature oocytes	14.9 (5.2)				Number of mature oocytes	15.6 (5.2)	
Engmann et al. (2008) (23)	USA	RCT – High risk (PCOS / previous high response)	GnRHa trigger	Leuprolide 1 mg	CPR	17	30	hCG trigger	3 000–10 000 IU	CPR	15	29
					Number of oocytes	20.2 (9.9)				Number of oocytes	18.8 (6.3)	
					OHSS	0				OHSS	10	
					Miscarriage	3				Miscarriage	4	
					Number of mature oocytes	24 (16.3)				Number of mature oocytes	19 (6.3)	
Engmann et al. (2019) (38)	USA	Double-blind RCT – High responders	Dual trigger	GnRHa (Leuprolide 1 mg) + hCG 1 000 IU (simultaneous administration)	CPR	15	26	GnRHa only	Leuprolide acetate 1 mg hCG 1 500 IU 35 h later for luteal support	CPR	33	31
					Number of oocytes	18.4 (5.9)				Number of oocytes	18.4 (7.7)	
					Miscarriage	5				Miscarriage	6	
Humaidan et al. (2013) (51)	Denmark / Belgium / Greece	Multicentre RCT – patients at high OHSS risk	GnRHa trigger	Buserelin 0.5 mg + 1 500 IU hCG post-retrieval for luteal support	CPR	21	60	hCG trigger	5 000 IU	CPR	17	58
					Number of oocytes	13.7 (5.9)				Number of oocytes	13.5 (5.7)	
					OHSS	0				OHSS	2	
					Miscarriage	4				Miscarriage	4	
Valipour et al. (2022) (54)	Iran	RCT – High risk (antagonist cycles)	Dual trigger	Decapeptyl 0.2 mg + hCG 2 500 IU (simultaneous)	CPR	32	50	hCG trigger	10 000 IU	CPR	16	50
					Number of oocytes	10.4 (4.1)				Number of oocytes	8.7 (3.3)	
					OHSS	0				OHSS	3	

SD, standard deviation; GnRHa, gonadotropin-releasing hormone agonist; hCG, human chorionic gonadotropin; OHSS, ovarian hyperstimulation syndrome; PCO(S), polycystic ovary (syndrome); CP, clinical pregnancy rate; OPR, ongoing pregnancy rate; LBR, live birth rate; NS, not significant.

Four studies totaling 395 participants compared GnRHa trigger to hCG trigger (22, 23, 41, 51), one study totaling 100 participants compared dual trigger to hCG trigger (54) and two studies consisting of 137 women compared GnRHa trigger to dual trigger (37, 38). There was no published RCT assessing the use of double trigger in women defined as being high responders.

### Risk of bias assessment

The risk of bias observed in the included studies ranged from low to high based on the Cochrane risk-of-bias tool. The quality assessment of the included studies is illustrated in **Supplementary Figure 1**.

### Network and pairwise meta-analyses

#### Laboratory outcomes

##### Number of oocytes

Five studies representing 414 participants reporting on number of oocytes retrieved were identified as being of high integrity and included (23, 37, 51, 54, 57). Pair wise meta-analysis, network plot and the results of the network meta-analysis are displayed in **Supplementary File 1**.

##### GnRH agonist trigger vs hCG trigger

Two studies compared GnRH agonist vs. hCG trigger in 177 participants (23, 51). There was insufficient evidence of a difference based on pairwise meta-analysis (MD 0.44, 95% CI: -1.44 to 2.31, I^2^ = 0%) and network meta-analysis (MD 1.08, 95% CI: -1.06 to 3.22). *Post hoc* power analysis yielded a critical z-value of approximately 1.97 and an achieved power of only 7.41%.

##### Dual trigger vs hCG trigger

One study compared dual vs. hCG trigger in 100 participants (54). There was insufficient evidence of a difference based on pairwise analysis (MD 1.70, 95% CI:0.24 to 3.16) and network meta-analysis (MD 0.61. 95% CI -1.53 to 2.74).

##### GnRH agonist trigger vs dual trigger

Two studies of 137 participants compared GnRH agonist vs. dual trigger (37, 57). There was insufficient evidence of a difference based on pairwise analysis (MD 0.97, 95% CI: -1.62 to 3.56, I^2^ = 0%) and network meta-analysis (MD 1.08. 95% CI -1.06 to 3.22). *Post hoc* power analysis yielded a critical z-value of approximately 1.97 and an achieved power of only 11.7%.

In the network meta-analysis, GnRH agonist trigger ranked highest for number of oocytes (SUCRA 76.1%), followed by dual trigger (51.5%) and hCG trigger (22.5%).

#### Number of matured oocytes

**Figure 2:**
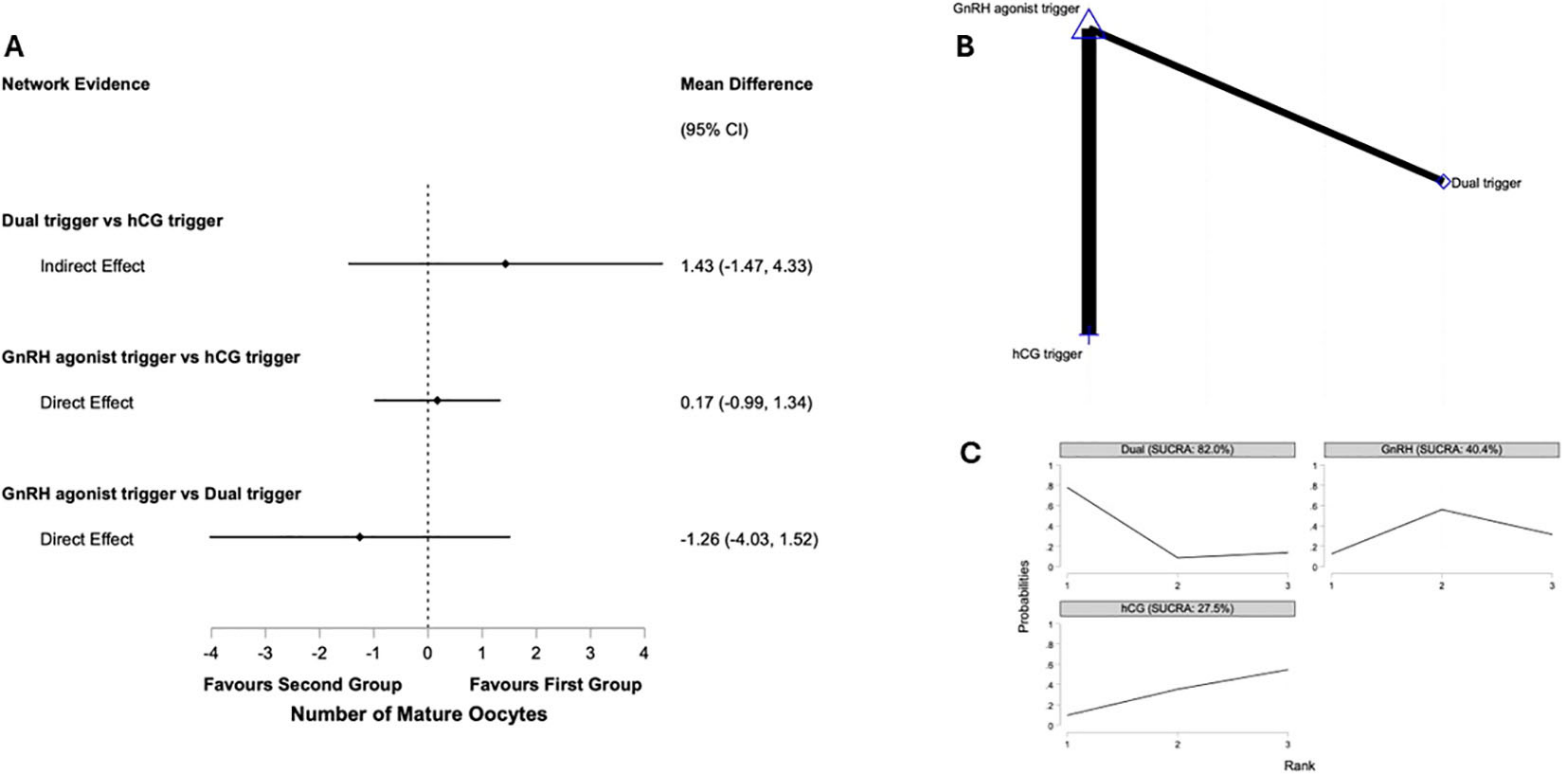
Network meta-analysis of number of mature oocytes in predicted high responders undergoing IVF antagonist cycles. **(A)** *Network forest plot* showing mean differences (MD) with 95% confidence intervals for number of mature oocytes between interventions. Positive values favour the first-listed intervention. **(B)** *Network geometry* illustrating direct and indirect evidence comparing final oocyte-maturation triggers: human chorionic gonadotropin (hCG), gonadotropin-releasing hormone agonist (GnRHa), and dual trigger (GnRHa low-dose hCG). Node size is proportional to the number of participants assigned to each intervention, and line thickness represents the number of trials providing direct comparisons. **(C)** *SUCRA ranking curves* displaying the relative probability of each intervention ranking among the most effective for number of mature obcytes.

Six studies representing 532 participants reporting on number of mature oocytes retrieved were identified as being of high integrity and included (22, 23, 37, 38, 41, 51). **Figure 2** presents the network plot for number of mature oocytes retrieved and the results of the network meta-analysis. Pair wise meta-analysis is displayed in **Supplementary File 1**.

##### GnRH agonist trigger vs hCG trigger

Four studies compared GnRH agonist vs. hCG trigger in 395 participants (22, 23, 41, 51). There was insufficient evidence of a difference based on pairwise meta-analysis (MD 1.11, 95% CI: -1.74 to 3.97, I^2^ = 76%, p=0.02) and network meta-analysis (MD -1.26, 95% CI: -4.03 to 1.52). *Post hoc* power analysis yielded a critical z-value of approximately 1.96 and an achieved power of only 12.5%.

##### Dual trigger vs hCG trigger

Through indirect evidence only, there was no difference in number of mature oocytes retrieved (MD 1.43, 95% CI: -1.47 to 4.33).

##### GnRH agonist trigger vs dual trigger

Two studies compared GnRH agonist vs. dual trigger in 137 participants (37, 38). There was insufficient evidence of a difference based on pairwise meta-analysis of these studies (MD 6.01, 95% CI: -2.40 to 14.41, I^2^ = 80%, p=0.03) and network meta-analysis (MD -1.26, 95% CI: -4.03 to 1.52). *Post hoc* power analysis yielded a critical z-value of approximately 1.97 and an achieved power of only 29.1%.

In the network meta-analysis, dual trigger ranked highest for number of mature oocytes (SUCRA 82.0%), followed by GnRH agonist (SUCRA 40.4%) and hCG trigger (SUCRA 27.5%).

#### Clinical outcomes

##### Clinical pregnancy rates

**Figure 3:**
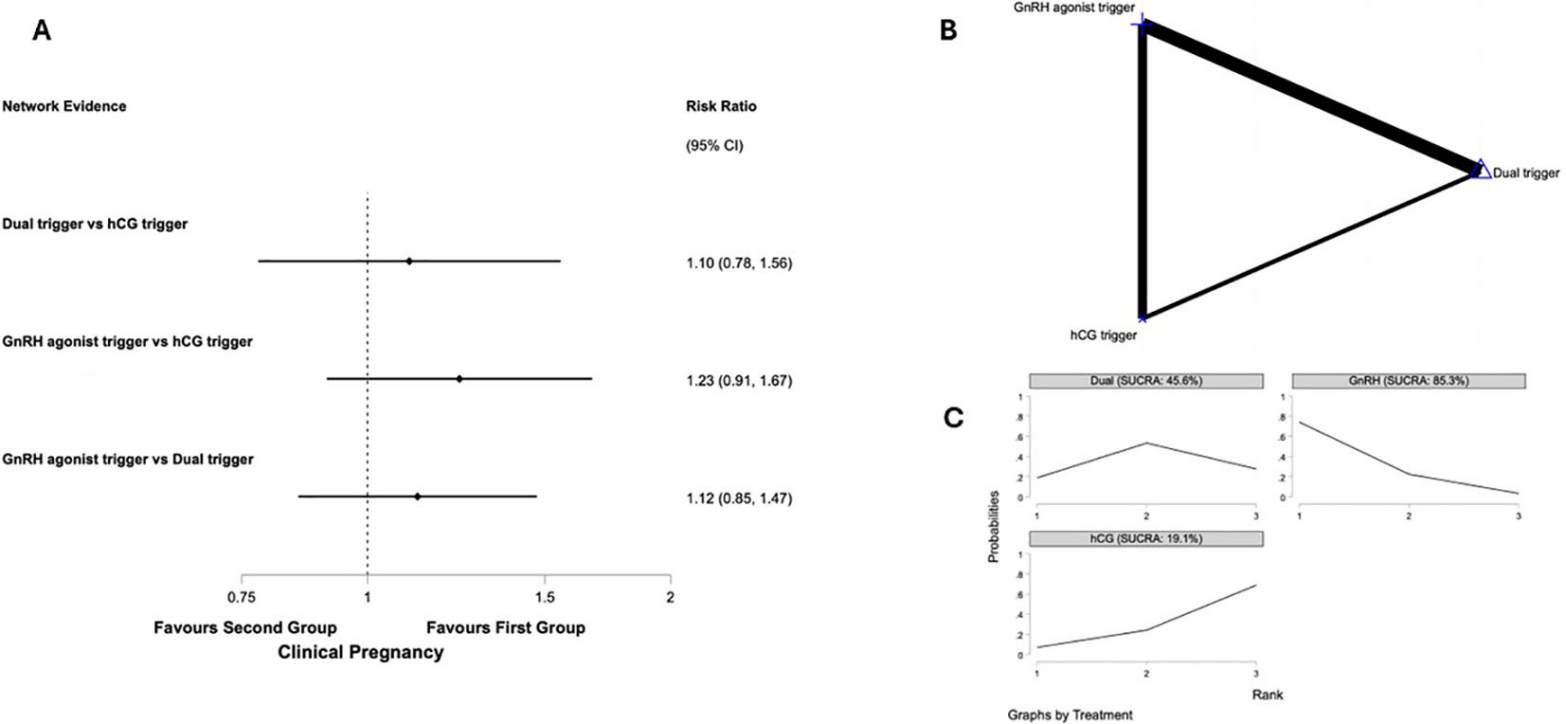
Network meta-analysis of clinical pregnancy rates in predicted high responders undergoing IVF antagonist cycles. **(A)** *Network forest plot* showing risk ratios (RR) with 95% confidence intervals for clinical pregnancy across interventions. Positive values favour the first-listed intervention. **(B)** *Network geometry* illustrating the evidence base comparing human chorionic gonadotropin (hCG), gonadotropin-releasing hormone agonist (GnRHa), and dual trigger (GnRHa + low-dose hCG) for final oocyte maturation. Node size corresponds to the total number of participants randomised to each intervention, and edge thickness represents the number of direct comparisons available between treatments. **(C)** *SUCRA ranking curves* displaying the relative probability of each intervention ranking among the highest for clinical pregnancy rates.

Five studies representing 414 participants reporting on clinical pregnancy rates (CPR) (23, 37, 51, 54, 57). **Figure 3** presents the network plot for clinical pregnancy rates and the results of the network meta-analysis. Pair wise meta-analysis is displayed in **Supplementary File 1**.

##### GnRH agonist trigger vs hCG trigger

Two studies compared GnRH agonist vs. hCG trigger in 177 participants (23, 51). There was insufficient evidence of a difference in CPR based on pairwise analysis (RR 1.14, 95% confidence interval (CI):0.80 to 1.62, I^2^ = 0%, p=0.81) and network meta-analysis (RR 1.23, 95% CI: 0.91 to 1.67). *Post hoc* power analysis yielded a critical z-value of approximately 1.96 and an achieved power of only 10.7%.

##### Dual trigger vs hCG trigger

One study compared dual vs. hCG trigger in 100 participants (54). There was insufficient evidence of a difference in CPR based on pairwise analysis (RR 1.31, 95% CI:0.78 to 2.21 p=0.30) and network meta-analysis (RR 1.100. 95% CI 0.78 to 1.56).

##### GnRH agonist trigger vs dual trigger

Two studies of 137 participants compared GnRH agonist vs. dual trigger (37, 57). There was insufficient evidence of a difference in CPR based on pairwise analysis (RR 1.15, 95% CI:0.85 to 1.57, I^2^ = 0%) and network meta-analysis (RR 1.12. 95% CI 0.85 to 1.47). *Post hoc* power analysis yielded a critical z-value of approximately 1.96 and an achieved power of only 14.2%.

In the network meta-analysis, GnRH agonist trigger ranked highest for CPR (SUCRA 85.3%), followed by dual trigger (45.6%) and hCG trigger (19.1%).

#### Miscarriage rates

Four studies representing 256 participants reporting on miscarriage rates were identified as being of high integrity and included (22, 23, 38, 51). Pair wise meta-analysis and network plot for rates of miscarriage and the results of the network meta-analysis are displayed in **Supplementary File 1**.

##### GnRH agonist trigger vs hCG trigger

Three studies compared GnRH agonist vs. hCG trigger in 277 participants (22, 23, 51). There was insufficient evidence of a difference in miscarriage rates based on pairwise meta-analysis of these studies (RR 0.85, 95% CI:0.36 to 2.00, I^2^ = 0%, p=0.71) and network meta-analysis (RR 0.85, 95% CI: 0.36,2.00). *Post hoc* power analysis yielded a critical z-value of approximately -1.96 and an achieved power of only 7.96%.

##### Dual trigger vs hCG trigger

Through indirect evidence only, there was no difference in miscarriage rates (RR 0.65. 95% CI 0.10 to 4.35).

##### GnRH agonist trigger vs dual trigger

One study compared GnRH agonist vs. dual trigger in 80 participants. There was insufficient evidence of a difference in miscarriage rates based on pairwise analysis (RR 2.52, 95% CI:0.28 to 22.76, p=0.41) and network meta-analysis (RR 1.31. 95% CI 0.24 to 7.14).

In the network meta-analysis, dual trigger ranked highest for rates of miscarriage (SUCRA 65.3%), followed by GnRH agonist (SUCRA 51.0%) and hCG trigger (SUCRA 33.7%).

#### Clinical safety

##### Moderate to severe ovarian hyperstimulation syndrome

**Figure 4:**
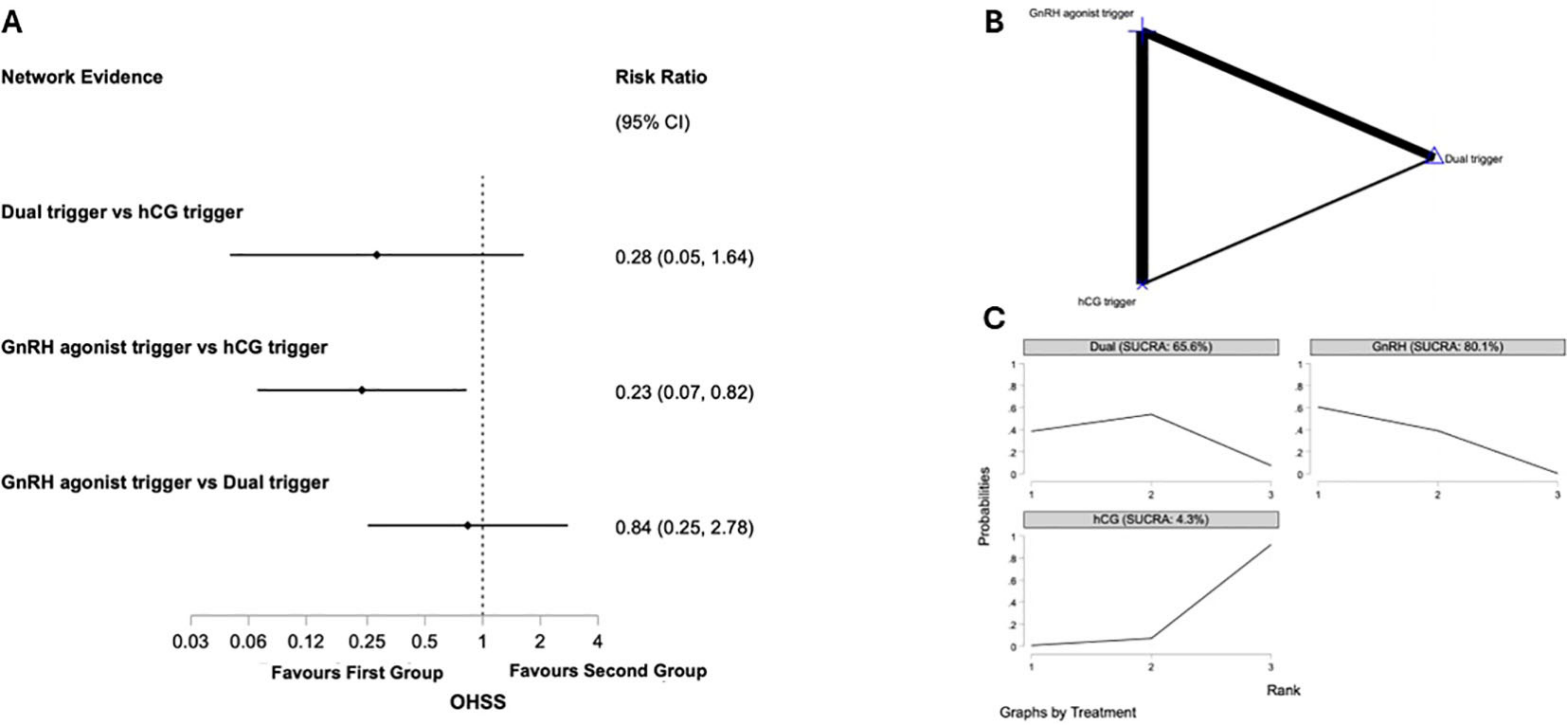
Network meta-analysis of rates of ovarian hyerstimulation (OHSS) in predicted high responders undergoing IVF antagonist cycles. **(A)** *Network forest plot* showing risk ratios (RR) with 95% confidence intervals for moderate to severe OHSS. Values <1 favour the first intervention **(B)** *Network geometry* depicting direct and indirect evidence comparing human chorionic gonadotropin (hCG), gonadotropin-releasing hormone agonist (GnRHa), and dual trigger (GnRHa low-dose hCG) for final oocyte maturation. Node size reflects the number of participants randomised to each Intervention, while the width of connecting lines indicates the number of trials contributing direct evidence. **(C)** *SUCRA ranking curves* displaying the relative probability of each intervention ranking among the highest OHSS rates.

Six studies representing 575 participants reporting on rates of OHSS were identified as being of high integrity and included (22, 23, 37, 41, 51, 54). **Figure 4** presents the network plot for moderate to severe OHSS rates and the results of the network meta-analysis. Pair wise meta-analysis is displayed in **Supplementary File 1**.

##### GnRH agonist trigger vs hCG trigger

Four studies compared GnRH agonist vs. hCG trigger in 395 participants (22, 23, 41, 51). The use of GnRH agonist was associated with lower risk of OHSS through pairwise meta-analysis (RR 0.25, 95% CI: 0.09 to 0.72 I^2^ = 21%, p=0.28) and network meta-analysis (RR 0.23, 95% CI: 0.07 to 0.82). *Post hoc* power analysis yielded a critical z-value of approximately 1.96 and an achieved power of only 99.6%.

##### Dual trigger vs hCG trigger

One study compared dual vs. hCG trigger in 100 participants (54). There was insufficient evidence of a difference based on pairwise analysis (RR 0.20. 95% CI 0.01 to 4.06) and network meta-analysis (RR 0.28. 95% CI 0.05 to 1.64).

##### GnRH agonist trigger vs dual trigger

Two studies compared GnRH agonist vs. dual trigger in 107 participants (37, 57). There was insufficient evidence of a difference based on pairwise analysis (RR 0.75. 95% CI 0.13 to 4.40, p=0.29) and network meta-analysis (RR 0.28. 95% CI 0.05 to 1.64). *Post hoc* power analysis yielded a critical z-value of approximately -1.96 and an achieved power of only 30.1%.

In the network meta-analysis, GnRH agonist trigger ranked highest for OHSS (SUCRA 80.1%), followed by followed by dual trigger (SUCRA 65.6%) and hCG trigger (SUCRA 4.3%).

## Discussion

In this network meta-analysis, we found no evidence of superiority among any trigger strategy for oocyte yield, oocyte maturity, clinical pregnancy, or miscarriage rates. The GnRH agonist trigger, however, was consistently associated with the lowest incidence of moderate to severe OHSS across studies. This observation aligns with the physiological rationale that GnRH agonist induces a brief, self-limited endogenous LH and FSH surge, thereby reducing the prolonged luteotropic stimulation and vascular permeability associated with hCG exposure (16).

Our findings challenge the assumption that GnRH agonist trigger inherently yield a higher number of mature oocytes due to the concurrent FSH surge (19, 20). In predicted high responders, the large follicular cohort and robust endogenous LH receptor expression may minimize any incremental benefit from exogenous stimulation. The comparable results observed across hCG, GnRH agonist, and dual triggers likely reflect convergence of downstream signaling pathways once the LH/hCG receptor is activated, irrespective of the initiating ligand Both hCG and LH activate the same receptor on granulosa and theca cells, initiating cyclic AMP–protein kinase A and ERK1/2 cascades that drive cumulus expansion, meiosis resumption, and luteinization although hCG has a longer half-life and stronger luteotropic effect, these pharmacodynamic differences appear to have limited clinical impact in this population (4, 8). Similarly, adding low-dose hCG in a dual-trigger protocol may not substantially enhance follicular competence beyond that achieved by the GnRH agonist-induced physiological FSH surge (5, 17, 58). The absence of differences in mature oocyte numbers supports the notion that all three protocols are equally effective in promoting oocyte maturation in high responders.

The timing of oocyte retrieval, typically standardized at 34–36 hours post-trigger, may also minimize kinetic variations between regimens (8, 19). Collectively, these biological and procedural overlaps likely explain why the three trigger modalities yielded comparable numbers of retrieved and mature oocytes, similar embryo quality, and equivalent clinical pregnancy rates (23, 27, 51, 59).

This study is, to our knowledge, the first network meta-analysis to synthesize evidence across all three trigger types in predicted high responders. By integrating direct and indirect comparisons, we provide a more comprehensive overview than previous pairwise analyses. Our selective focus on high-integrity trials strengthens the validity of our findings, ensuring that our conclusions are based on the most reliable and scientifically robust evidence available and the innovative aspect of our study is its comprehensive comparative analysis of all three types of final oocyte maturation triggers using a NMA framework. Unlike previous studies that primarily focused on direct head-to-head comparisons between two techniques, our NMA approach provides a more extensive evaluation. By simultaneously assessing the three trigger protocols, we aim to fill a critical gap in the existing literature.

Nonetheless, several limitations warrant consideration. While we aimed to perform a comparison of triggering options including the use of double trigger, this was not possible due to the lack of published RCTs. Secondly, the studies included in this meta-analysis often had small sample sizes, which may have affected the precision and generalizability of our findings. Live birth rate was also prespecified as a primary outcome in the PROSPERO-registered protocol; however, this outcome could not be meta-analyzed due to no reporting across the included trials. This represents a deviation from the original protocol, and the decision to exclude this outcome from quantitative synthesis was made *a priori* during data extraction. Our *post hoc* power analyses also consistently revealed low power across the comparisons, indicating that the current sample sizes are insufficient to reliably detect the small differences observed between the trigger strategies. This underpowering increases the risk of Type II errors, meaning that a true effect may remain undetected due to the small sample size of published RCTs included in the analysis. Consequently, when our meta-analysis did not find statistically significant differences between the compared groups, these null results should be interpreted with caution. The limited power suggests that the absence of evidence does not necessarily equate to evidence of absence. Equally, while the GnRH agonist trigger also had the highest SUCRA ranking for several outcomes, these rankings should be interpreted with caution with the understanding that this ranking reflects relative probabilities of being the most effective treatment rather than definitive evidence of superiority, and their reliability diminishes in sparse networks with few trials and wide confidence intervals. The small number of included studies and low achieved power further limit the precision of comparative estimates.

When treating patients defined as high responders, clinicians are acutely aware of the risk of OHSS, and the use of GnRH agonist trigger is therefore often preferred. Given the documented reduction in OHSS and comparable efficacy, many centers increasingly favor a freeze-all strategy following GnRH agonist triggering. However, some patients may still opt for fresh embryo transfer due to financial or logistical reasons, and not all IVF programs achieve equivalent frozen transfer outcomes. Offering an oocyte maturation trigger option which facilitates a fresh embryo transfer while continuing to benefit from the advantages of GnRH agonist trigger in reducing the risk of ovarian hyperstimulation syndrome (OHSS) is therefore of clinical interest to many.

Overall, trigger selection should be individualized based on ovarian response, previous cycle characteristics, and transfer strategy (fresh vs. frozen). The double-trigger approach, although conceptually appealing, remains underexplored in high responders and warrants further study to define its role in optimizing both oocyte maturation and luteal competence and our findings highlight the need for well-powered RCTs directly comparing GnRH agonist, dual, and double triggers, with live birth as a primary endpoint, consistent with the fertility research core outcome set (44, 45). While the dual trigger was not seen to be superior for oocyte yield, mature oocyte yield of reproductive outcomes, patients who may particularly benefit from a dual-trigger strategy include individuals planning a fresh embryo transfer where luteal support is critical.

Future research should include larger patient populations and harmonized outcome definitions to improve precision and comparability across trials. Expanding the evidence base will be essential to clarify whether subtle differences in trigger type translate into clinically meaningful improvements in IVF outcomes for high-responder patients.

## Conclusion

Our network meta-analysis evaluated the use of three different final oocyte maturation trigger protocols in predicted high responder patients undergoing IVF/ICSI within GnRH antagonist cycles. Our findings suggest comparable efficacy among hCG, GnRH agonist, and dual trigger protocols in terms of clinical pregnancy rates and oocyte yield. Notably, the GnRH agonist trigger remains safest and significantly reduced ovarian hyperstimulation syndrome risk compared to hCG, further reinforcing its use in predicted high responder patients.

Our findings support using GnRH agonist triggers to reduce OHSS risk without compromising pregnancy outcomes, given intensified luteal phase support. However, the dual trigger’s comparable efficacy may be considered as a balanced alternative for some patients.

This study highlights the importance of personalized medicine approaches in IVF/ICSI treatment and study emphasizes the need for further research, particularly large-scale randomized controlled trials evaluating live birth outcomes across all trigger protocols.

## Data Availability

The original contributions presented in the study are included in the article/**Supplementary Material**. Further inquiries can be directed to the corresponding author.
