# Supplementary material for: Oocyte maturation triggering in high responders in IVF treatment: a systematic review and network meta-analysis
Source: Front Endocrinol (Lausanne). 2026 Apr 2;17:1669781. doi: 10.3389/fendo.2026.1669781 (PMC13082988; doi:10.3389/fendo.2026.1669781)
Supplement: Supplementary file 1 [file SupplementaryFile1.docx]

| **Number of oocytes in high responder**  **Supplementary Table 1. CINeMA assessment of confidence in direct and indirect evidence for all pairwise comparisons of trigger strategies.** | | | |
| --- | --- | --- | --- |
| Intervention compared | Direct evidence | Indirect evidence | Network Analysis |
| GnRH agonist trigger vs hCG trigger | MD -1.33 (95% CI: -4.02-1.35)  4 trials (401 participants)  ⨁⨁◯◯  Low | - | MD -1.33 (95% CI: -4.02-1.35)  ⨁⨁◯◯  Low |
| Dual trigger vs hCG trigger | MD 1.28 (95% CI: -1.54-4.10)  3 trials (448 participants)  ⨁⨁◯◯  Low | - | MD 1.28 (95% CI: -1.54-4.10)  3 trials (448 participants)  ⨁⨁◯◯  Low |
| Double trigger vs hCG trigger | - | MD 0.26 (95% CI: -4.84-5.36)  ⨁◯◯◯  Very low | MD 0.26 (95% CI: -4.84-5.36)  ⨁◯◯◯  Very low |
| Dual trigger vs GnRH agonist trigger | - | MD 2.61 (95% CI: -1.28-6.51)  ⨁◯◯◯  Very low | MD 2.61 (95% CI: -1.28-6.51)  ⨁◯◯◯  Very low |
| Double trigger vs GnRH agonist trigger | - | MD 1.59 (95% CI: -4.17-7.36)  ⨁◯◯◯  Very low | MD 1.59 (95% CI: -4.17-7.36)  ⨁◯◯◯  Very low |
| Double trigger vs Dual trigger | MD -1.02 (95% CI: -5.27-3.23)  2 trials (137 participants)  ⨁◯◯◯  Very low | - | MD -1.02 (95% CI: -5.27-3.23)  ⨁◯◯◯  Very low |
| **Number of mature oocytes in high responder** | | | |
| Intervention compared | Direct evidence | Indirect evidence | Network Analysis |
| GnRH agonist trigger vs hCG trigger | MD 1.46 (95% CI: -0.46-3.38)  6 trials (645 participants)  ⨁◯◯◯  Very low | - | MD 1.46 (95% CI: -0.46-3.38)   ⨁◯◯◯  Very low |
| Dual trigger vs hCG trigger | MD 0.22 (95% CI: -2.16-2.61)  2 trials (236 participants)  ⨁◯◯◯  Very low | - | MD 0.22 (95% CI: -2.16-2.61)  ⨁◯◯◯  Very low |
| Double trigger vs hCG trigger | - | MD -1.68 (95% CI: -6.67-3.31)  ⨁◯◯◯  Very low | MD -1.68 (95% CI: -6.67-3.31)  ⨁◯◯◯  Very low |
| Dual trigger vs GnRH agonist trigger | - | MD -1.23 (95% CI: -4.30-1.83)  ⨁◯◯◯  Very low | MD -1.23 (95% CI: -4.30-1.83)  ⨁◯◯◯  Very low |
| Double trigger vs GnRH agonist trigger | - | MD -3.13 (95% CI: -8.48-2.22)  ⨁◯◯◯  Very low | MD -3.13 (95% CI: -8.48-2.22)  ⨁◯◯◯  Very low |
| Double trigger vs Dual trigger | MD -1.90 (95% CI: -6.28-2.48)  1 trial (80 participants)  ⨁◯◯◯  Very low | - | MD -1.90 (95% CI: -6.28-2.48)  ⨁◯◯◯  Very low |
| **Clinical pregnancy in high responder** | | | |
| Intervention compared | Direct evidence | Indirect evidence | Network Analysis |
| GnRH agonist trigger vs hCG trigger | RR 1.04 (95% CI: 0.84-1.29)  4 trials (424 participants)  ⨁⨁◯◯  Low |  | RR 1.04 (95% CI: 0.84-1.29)  ⨁⨁◯◯  Low |
| Dual trigger vs hCG trigger | RR 1.82 (95% CI: 1.25-2.67)  2 trials (220 participants)  ⨁⨁◯◯  Low |  | RR 1.82 (95% CI: 1.25-2.67)  ⨁⨁◯◯  Low |
| Double trigger vs hCG trigger |  | RR 2.10 (95% CI: 1.29-3.43)  ⨁⨁◯◯  Low | RR 2.10 (95% CI: 1.29-3.43)  ⨁⨁◯◯  Low |
| Dual trigger vs GnRH agonist trigger |  | RR 1.76 (95% CI: 1.14-2.72)  ⨁⨁◯◯  Low | RR 1.76 (95% CI: 1.14-2.72)  ⨁⨁◯◯  Low |
| Double trigger vs GnRH agonist trigger |  | RR 2.03 (95% CI: 1.19-3.46)  ⨁⨁◯◯  Low | RR 2.03 (95% CI: 1.19-3.46)  ⨁⨁◯◯  Low |
| Double trigger vs Dual trigger | RR 1.15 (95% CI: 0.85-1.57)  1 trial (80 participants)  ⨁⨁◯◯  Low |  | RR 1.15 (95% CI: 0.85-1.57)  ⨁⨁◯◯  Low |
| **Ongoing pregnancy in high responder** | | | |
| Intervention compared | Direct evidence | Indirect evidence | Network Analysis |
| GnRH agonist trigger vs hCG trigger | RR 1.10 (95% CI: 0.75-1.62)  3 trials (234 participants)  ⨁⨁◯◯  Low |  | RR 1.10 (95% CI: 0.75-1.62)    ⨁⨁◯◯  Low |
| Dual trigger vs hCG trigger | No evidence available | No evidence available | No evidence available |
| Double trigger vs hCG trigger | No evidence available | No evidence available | No evidence available |
| Dual trigger vs GnRH agonist trigger | No evidence available | No evidence available | No evidence available |
| Double trigger vs GnRH agonist trigger | No evidence available | No evidence available | No evidence available |
| Dual trigger vs Double trigger | RR 0.94 (95% CI: 0.61-1.45)  1 trial (XX participants)  ⨁⨁◯◯  Low |  | RR 0.94 (95% CI: 0.61-1.45)    ⨁⨁◯◯  Low |
| **Live birth in high responder** | | | |
| Intervention compared | Direct evidence | Indirect evidence | Network Analysis |
| GnRH agonist trigger vs hCG trigger | RR 0.43 (95% CI: 0.04-5.35)  2 trials (914 participants)  ⨁◯◯◯  Very low |  | RR 0.43 (95% CI: 0.04-5.35)  ⨁◯◯◯  Very low |
| Dual trigger vs hCG trigger | No evidence available | No evidence available | No evidence available |
| Double trigger vs hCG trigger | No evidence available | No evidence available | No evidence available |
| Dual trigger vs GnRH agonist trigger | No evidence available | No evidence available | No evidence available |
| Double trigger vs GnRH agonist trigger | No evidence available | No evidence available | No evidence available |
| Double trigger vs Dual trigger | RR 0.88 (95% CI: 0.23-3.43)  1 trial (57 participants)  ⨁◯◯◯  Very low |  | RR 0.88 (95% CI: 0.23-3.43)   ⨁◯◯◯  Very low |
| **Miscarriage in high responder** | | | |
| Intervention compared | Direct evidence | Indirect evidence | Network Analysis |
| GnRH agonist trigger vs hCG trigger | RR 0.85 (95% CI: 0.36-2.00)  3 trials (205 participants)  ⨁⨁◯◯  Low |  | RR 0.85 (95% CI: 0.36-2.00)   ⨁⨁◯◯  Low |
| Dual trigger vs hCG trigger | No evidence available | No evidence available | No evidence available |
| Double trigger vs hCG trigger | No evidence available | No evidence available | No evidence available |
| Dual trigger vs GnRH agonist trigger | No evidence available | No evidence available | No evidence available |
| Double trigger vs GnRH agonist trigger | No evidence available | No evidence available | No evidence available |
| Double trigger vs Dual trigger | RR 0.80 (95% CI: 0.28-2.29)  1 trial (31 participants)  ⨁⨁◯◯  Low |  | RR 0.80 (95% CI: 0.28-2.29)  ⨁⨁◯◯  Low |
| **Ovarian hyperstimulation in high responder** | | | |
| Intervention compared | Direct evidence | Indirect evidence | Network Analysis |
| GnRH agonist trigger vs hCG trigger | RR 0.23 (95% CI: 0.06-0.90)  5 trials (660 participants)  ⨁⨁⨁◯  Moderate |  | RR 0.23 (95% CI: 0.06-0.90)  ⨁⨁⨁◯  Moderate |
| Dual trigger vs hCG trigger | RR 0.59 (95% CI: 0.09-3.89)  2 trial (300 participants)  ⨁⨁◯◯  Low |  |  |
| Double trigger vs hCG trigger |  | RR 0.23 (95% CI: 0.02-2.64)  ⨁⨁◯◯  Low | RR 0.23 (95% CI: 0.02-2.64)  ⨁⨁◯◯  Low |
| Dual trigger vs GnRH agonist trigger |  | RR 2.54 (95% CI: 0.26-24.35)  ⨁⨁◯◯  Low | RR 2.54 (95% CI: 0.26-24.35)  ⨁⨁◯◯  Low |
| Double trigger vs GnRH agonist trigger |  | RR 0.98 (95% CI: 0.06-15.33)  ⨁⨁◯◯  Low | RR 0.98 (95% CI: 0.06-15.33)  ⨁⨁◯◯  Low |
| Double trigger vs Dual trigger | RR 0.38 (95% CI: 0.08-1.85)  1trial (80 participants)  ⨁⨁◯◯  Low |  | RR 0.38 (95% CI: 0.08-1.85)  ⨁⨁◯◯  Low |
