# Supplementary material for: Oocyte maturation triggering in high responders in IVF treatment: a systematic review and network meta-analysis
Source: Front Endocrinol (Lausanne). 2026 Apr 2;17:1669781. doi: 10.3389/fendo.2026.1669781 (PMC13082988; doi:10.3389/fendo.2026.1669781)
Supplement: Supplementary file 2 [file SupplementaryFile2.docx]

Supplementary Files

**Figure 1. Methodological quality summary: review authors' judgements about each methodological quality item for each included study.**


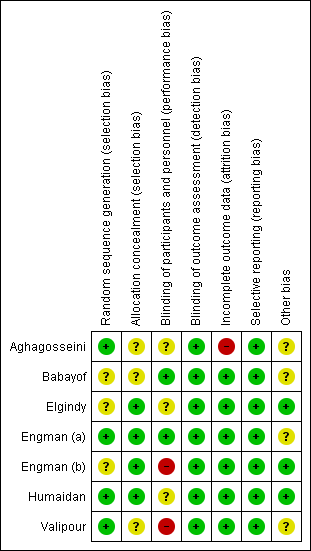

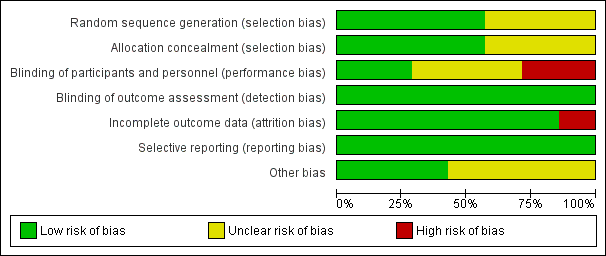


**Figure 2. Pairwise analysis** **for clinical pregnancy comparing final oocyte maturation trigger with GnRH agonist, hCG or dual trigger to each other, presented in RRs.**


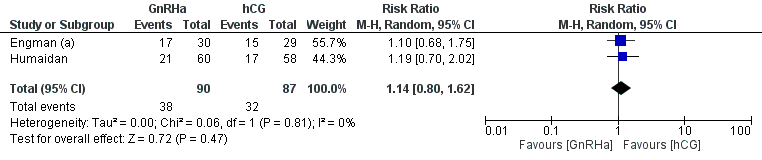


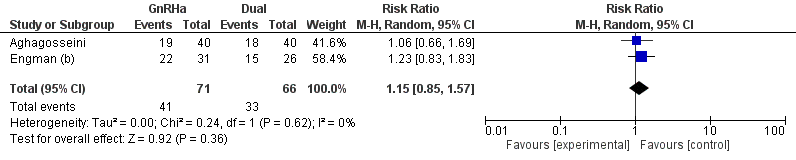


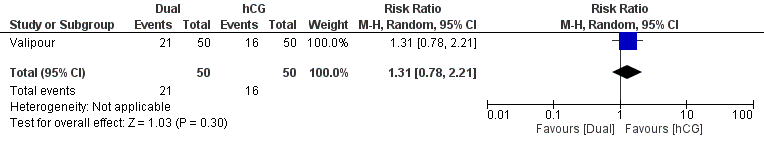


**Figure 3. Pairwise analysis** **for number of oocytes comparing final oocyte maturation trigger with GnRH agonist, hCG or dual trigger to each other, presented in MDs.**


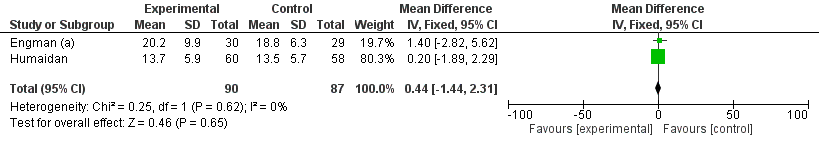


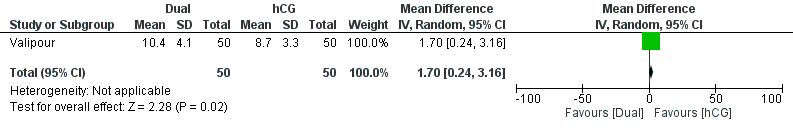

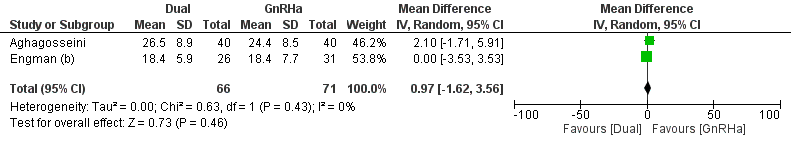


**Figure 4. Network map and meta-analysis and SUCRA ranking for number of oocytes comparing final oocyte maturation trigger with GnRH agonist, hCG or dual trigger to each other, presented in MDs**

**
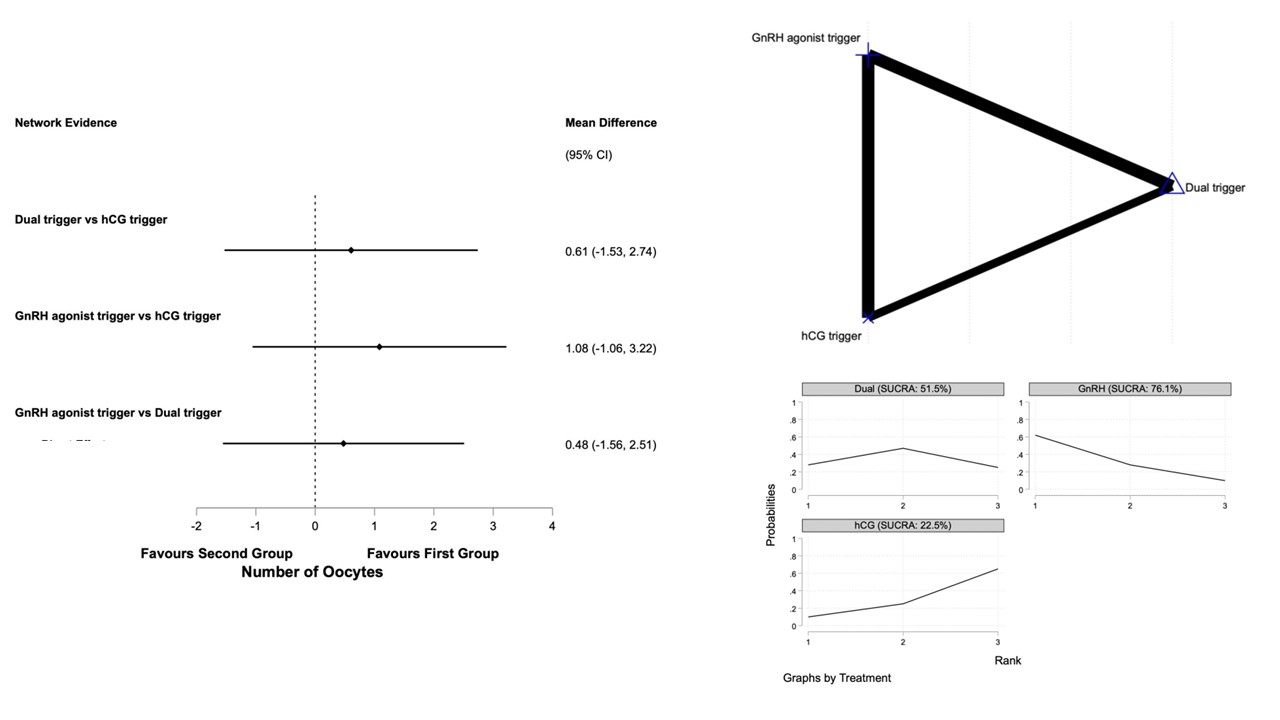
**

**Figure 5. Pairwise analysis** **for number of mature oocytes comparing final oocyte maturation trigger with GnRH agonist, hCG or dual trigger to each other, presented in MDs.**


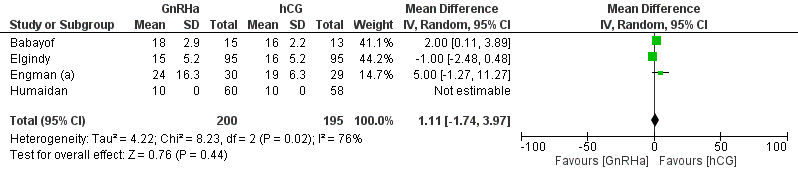


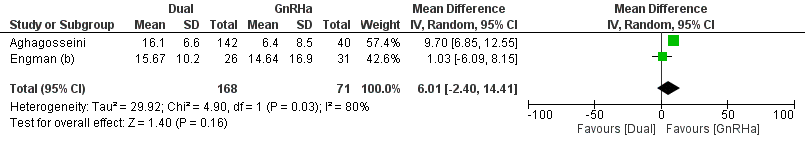


**Figure 6. Pairwise analysis** **for** **moderate to severe OHSS rates comparing final oocyte maturation trigger with GnRH agonist, hCG or dual trigger to each other, presented in RRs.**


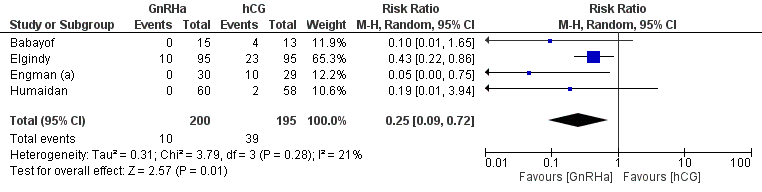


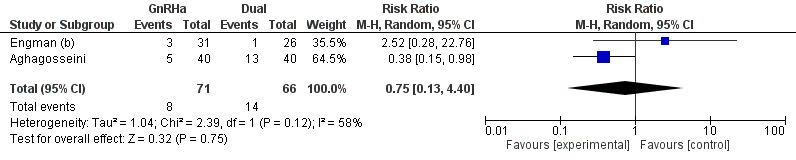


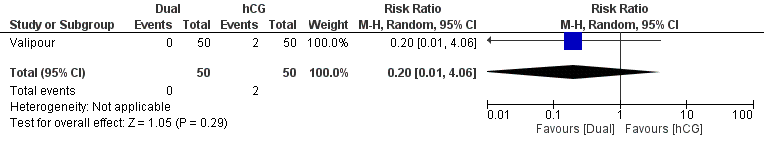


**Figure 7. Pairwise analysis** **for miscarriage rates comparing final oocyte maturation trigger with GnRH agonist, hCG or dual trigger to each other, presented in RRs.**


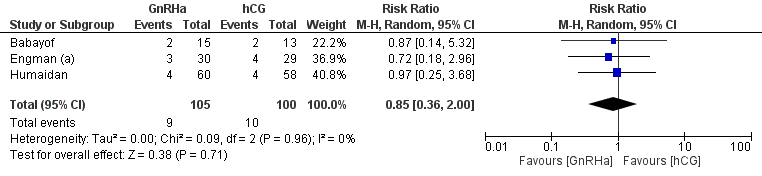


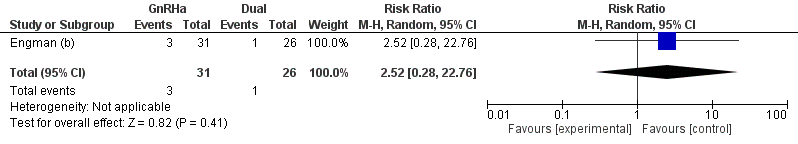


**Figure 8. Network map and meta-analysis and SUCRA ranking for miscarriage rates comparing final oocyte maturation trigger with GnRH agonist, hCG or dual trigger to each other, presented in RRs**

**
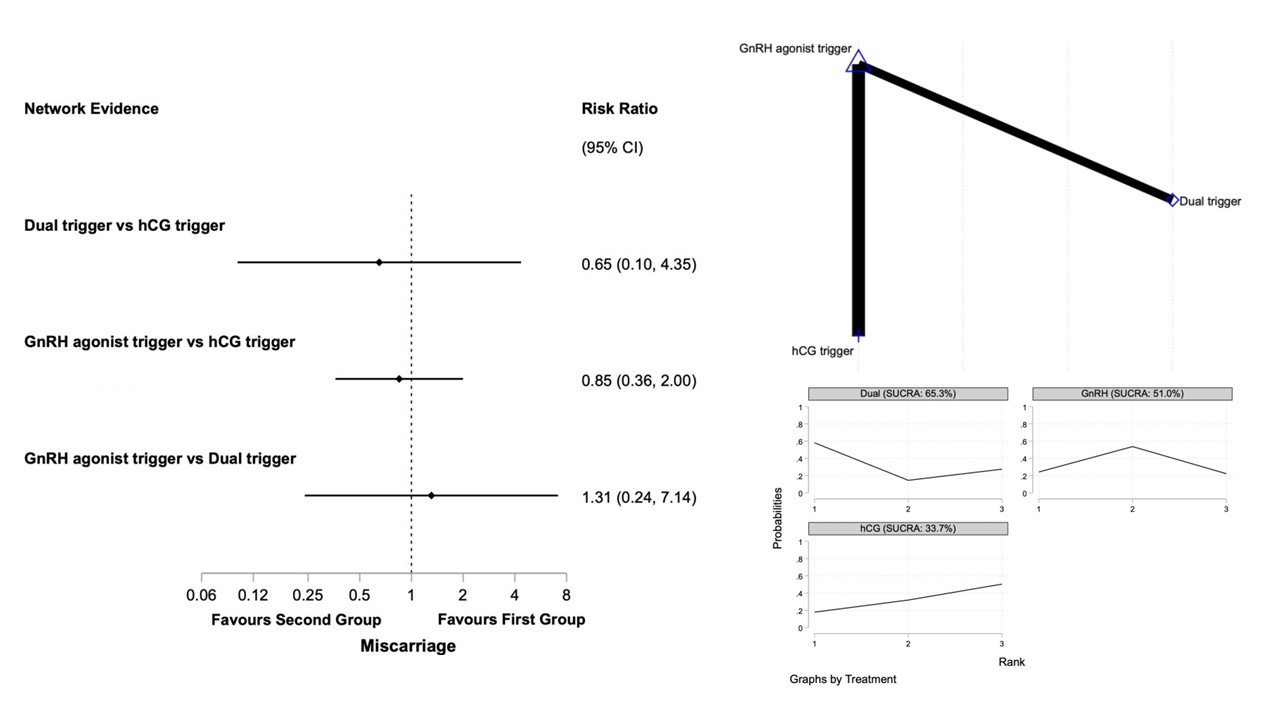
**
