# Supplementary material for: Oocyte maturation triggering in high responders in IVF treatment: a systematic review and network meta-analysis
Source: Front Endocrinol (Lausanne). 2026 Apr 2;17:1669781. doi: 10.3389/fendo.2026.1669781 (PMC13082988; doi:10.3389/fendo.2026.1669781)
Supplement: Supplementary file 3 [file SupplementaryFile3.docx]

**Search strategies**

**CENTRAL via the Cochrane Register of Studies Online (CRSO)***

Web platform

Searched on 30 December 2024

#1 MESH DESCRIPTOR Gonadotropin-Releasing Hormone EXPLODE ALL TREES 2707

#2 (gonadotropin-releasing hormone*):TI,AB,KY 2480

#3 (gonadotrophin releasing hormone*):TI,AB,KY 514

#4 (buserelin or Suprefact):TI,AB,KY 542

#5 (goserelin or Zoladex):TI,AB,KY 1176

#6 (leuprolide or lupron):TI,AB,KY 1095

#7 (nafarelin or Synarel):TI,AB,KY 146

#8 (histrelin or Supprelin):TI,AB,KY 8

#9 (deslorelin or Suprelorin or Ovuplant):TI,AB,KY 107

#10 triptorelin*:TI,AB,KY 904

#11 (GnRH agonist*):TI,AB,KY 1794

#12 (GnRH a):TI,AB,KY 425

#13 GnRHa:TI,AB,KY 534

#14 MESH DESCRIPTOR Chorionic Gonadotropin EXPLODE ALL TREES 917

#15 (chorionic gonadotropin*):TI,AB,KY 2184

#16 (chorionic gonadotrophin*):TI,AB,KY 438

#17 HCG*:TI,AB,KY 3400

#18 ?HCG*:TI,AB,KY 3526

#19 (pregnyl or Profasi or Ovidrel or Novarel or Chorex):TI,AB,KY 83

#20 #1 OR #2 OR #3 OR #4 OR #5 OR #6 OR #7 OR #8 OR #9 OR #10 OR #11 OR #12 OR #13 6080

#21 #14 OR #15 OR #16 OR #17 OR #18 OR #19 4357

#22 #20 OR #21 9158

#23 MESH DESCRIPTOR Oocyte Retrieval EXPLODE ALL TREES 209

#24 MESH DESCRIPTOR Ovulation Induction EXPLODE ALL TREES 1443

#25 (dual adj3 trigger*):TI,AB,KY 78

#26 (double adj3 trigger*):TI,AB,KY 41

#27 ((oocyte* or ovum or ovarian or ovary or ovaries or ovulat*) adj5 (matur* or collect* or retriev* or aspirat* or stimulat* or trigger* or induce or induction)):TI,AB,KY 6424

#28 ((oocyte* or ovum) adj3 competenc*):TI,AB,KY 50

#29 #23 OR #24 OR #25 OR #26 OR #27 OR #28 6500

#30 #22 AND #29 3074

*CENTRAL also contains trial registrations from two trial registries: ClinicalTrials.gov and the WHO International Clinical Trials Registry Platform (ICTRP).

**MEDLINE**

Ovid platform

Searched from 1946 to 30 December 2024

1 exp gonadotropin-releasing hormone/ or exp buserelin/ or exp goserelin/ or exp leuprolide/ or exp nafarelin/ or exp triptorelin/ (33786)

2 gonadotropin-releasing hormone$.tw. (15418)

3 (buserelin or Suprefact).tw. (1408)

4 (goserelin or Zoladex).tw. (1248)

5 (leuprolide or lupron).tw. (2113)

6 (nafarelin or Synarel).tw. (266)

7 (histrelin or Supprelin).tw. (78)

8 (deslorelin or Suprelorin or Ovuplant).tw. (320)

9 triptorelin$.tw. (862)

10 gonadotropin-releasing hormone agonist$.tw. (2590)

11 gonadotrophin releasing hormone agonist$.tw. (572)

12 GnRH agonist$.tw. (4832)

13 GnRH a.tw. (1175)

14 GnRHa.tw. (1790)

15 or/1-14 (41535)

16 exp Chorionic Gonadotropin/ (32437)

17 chorionic gonadotropin$.tw. (17411)

18 chorionic gonadotrophin$.tw. (4906)

19 HCG$.tw. (26814)

20 ?HCG.tw. (26189)

21 pregnyl.tw. (52)

22 Profasi.tw. (30)

23 Ovidrel.tw. (17)

24 Novarel.tw. (2)

25 or/16-24 (47040)

26 15 or 25 (84534)

27 oocyte retrieval/ or exp ovulation induction/ (15713)

28 trigger$.tw. (337261)

29 ((oocyte$ or ovum or ovarian or ovary or ovaries or ovulat$) adj5 (matur$ or collect$ or retriev$ or aspirat$ or stimulat$ or trigger$ or induce or induction)).tw. (46961)

30 (oocyte$ adj3 competence$).tw. (2067)

31 or/27-30 (387692)

32 26 and 31 (12890)

33 randomized controlled trial.pt. (575455)

34 controlled clinical trial.pt. (94990)

35 randomized.ab. (571999)

36 randomised.ab. (113784)

37 placebo.tw. (237155)

38 clinical trials as topic.sh. (200304)

39 randomly.ab. (389093)

40 trial.ti. (268631)

41 (crossover or cross-over or cross over).tw. (94579)

42 or/33-41 (1543274)

43 exp animals/ not humans.sh. (5039520)

44 42 not 43 (1420423)

45 32 and 44 (1924)

**Embase**

Ovid platform

Searched from 1 January 2020 to 30 December 2024

1 gonadorelin derivative/ or buserelin/ or buserelin acetate/ or deslorelin/ or folligen/ or exp gonadorelin/ or exp gonadorelin acetate/ or exp gonadorelin agonist/ or exp goserelin/ or exp histrelin/ or exp leuprorelin/ or exp lutrelin/ or exp nafarelin/ or exp nafarelin acetate/ or exp ovurelin/ or exp triptorelin/ (72169)

2 gonadorelin$.tw. (398)

3 gonadotropin-releasing hormone$.tw. (17474)

4 (buserelin or Suprefact).tw. (2524)

5 (goserelin or Zoladex).tw. (3394)

6 (leuprolide or lupron).tw. (4769)

7 (nafarelin or Synarel).tw. (636)

8 (histrelin or Supprelin).tw. (192)

9 (deslorelin or Suprelorin or Ovuplant).tw. (397)

10 triptorelin$.tw. (1472)

11 gonadotropin-releasing hormone agonist$.tw. (3175)

12 gonadotrophin releasing hormone agonist$.tw. (676)

13 GnRH agonist$.tw. (7533)

14 GnRH a.tw. (1513)

15 GnRHa.tw. (2693)

16 or/1-14 (76407)

17 exp chorionic gonadotropin/ (46151)

18 chorionic gonadotropin$.tw. (17957)

19 chorionic gonadotrophin$.tw. (4924)

20 HCG$.tw. (34987)

21 ?HCG.tw. (34150)

22 (pregnyl or Profasi or Ovidrel or Novarel or Chorex).tw. (3454)

23 or/17-22 (62121)

24 exp oocyte maturation/ or oocyte retrieval/ (20304)

25 trigger$.tw. (421687)

26 ((oocyte$ or ovum or ovarian or ovary or ovaries or ovulat$) adj5 (matur$ or collect$ or retriev$ or aspirat$ or stimulat$ or trigger$ or induce or induction)).tw. (63232)

27 (oocyte$ adj3 competence$).tw. (2776)

28 or/24-27 (485435)

29 16 or 23 (128459)

30 28 and 29 (20150)

31 Clinical Trial/ (1028357)

32 Randomized Controlled Trial/ (713851)

33 controlled clinical trial/ (466493)

34 multicenter study/ (329163)

35 Phase 3 clinical trial/ (61336)

36 Phase 4 clinical trial/ (4836)

37 exp randomization/ (94483)

38 Single Blind Procedure/ (46835)

39 Double Blind Procedure/ (193703)

40 Crossover Procedure/ (70809)

41 Placebo/ (369468)

42 Randomi?ed controlled trial$.tw. (291850)

43 Rct.tw. (47926)

44 (random$ adj2 allocat$).tw. (50189)

45 Single blind$.tw. (28878)

46 Double blind$.tw. (224674)

47 ((treble or triple) adj blind$).tw. (1578)

48 placebo$.tw. (340505)

49 prospective study/ (778122)

50 or/31-49 (2690941)

51 case study/ (86606)

52 case report.tw. (480762)

53 abstract report/ or letter/ (1197546)

54 Editorial.pt. (720763)

55 Letter.pt. (1199565)

56 Note.pt. (899064)

57 or/51-56 (3441047)

58 50 not 57 (2557129)

59 30 and 58 (4554)

Cochrane Gynaecology and Fertility (CGF) Group’s Specialised Register

ProCite database

Searched from inception to 23 November 2023 (the Specialised Register has not been updated past this date)

Keywords CONTAINS "trigger" or "triggered ovulation" or "ovulation trigger" or "oocyte maturation" or "double trigger" or "dual trigger" or Title CONTAINS "trigger" or "triggered ovulation" or "ovulation trigger" or "oocyte maturation" or "double trigger" or "dual trigger"

(371 records)
